# Emerging perspectives on the selective autophagy of melanosomes: melanophagy

**DOI:** 10.1038/s12276-025-01581-3

**Published:** 2025-11-14

**Authors:** Na Yeon Park, Seong Hyun Kim, Doo Sin Jo, Dong-Hyung Cho

**Affiliations:** 1 Organelle Institute, Kyungpook National University, Daegu, Republic of Korea; 2 School of Life Sciences, BK21 FOUR KNU Creative BioResearch Group, Kyungpook National University, Daegu, Republic of Korea; 3 ORGASIS Corporation, Suwon, Republic of Korea

**Keywords:** Macroautophagy, Autophagosomes, Ageing

## Abstract

Melanosomes are highly specialized organelles responsible for melanin synthesis, storage and transport in melanocytes, playing a central role in pigmentation and skin homeostasis. Although melanosome biogenesis and trafficking have been well characterized, emerging evidence emphasizes the importance of melanosome degradation in regulating pigment levels. Among the degradation pathways, melanophagy—a selective form of autophagy targeting melanosomes—has recently emerged as an important mechanism for the turnover of damaged, immature, or excess melanosomes. Here we highlight current insights into melanophagy mechanisms, including molecular regulators and signaling pathways. We also discuss the potential of modulating melanophagy as a novel cosmetic or therapeutic approach for managing hyperpigmentation, offering an alternative to traditional strategies focused solely on inhibiting melanin synthesis. By emphasizing the role of organelle clearance, melanophagy provides a new paradigm in the regulation of skin pigmentation.

## Melanophagy revealed as key to skin pigmentation control

The skin is our body’s largest organ, protecting us from environmental harm and helping with temperature control and sensation. Melanosomes are tiny organelles in skin cells that produce and store melanin, the pigment responsible for skin color. This article explores how melanosomes are broken down through a process of selective autophagy called melanophagy. Researchers have found that melanophagy helps maintain skin color by removing excess or damaged melanosomes. They used laboratory studies to understand how certain proteins and pathways regulate this process. For example, they discovered that specific proteins tag melanosomes for degradation, ensuring proper pigment balance. The study concludes that understanding melanophagy could lead to new treatments for pigmentation disorders such as vitiligo or hyperpigmentation. In the future, targeting melanophagy might offer safer ways to manage skin pigmentation without disrupting other cellular functions. This summary was initially drafted using artificial intelligence, then revised and fact-checked by the author.

## Introduction

The skin is the largest organ of the body and acts as a protective barrier against environmental stress, pathogens and water loss. It also contributes to immune defense, temperature regulation and sensory functions. Structurally, the skin consists of three layers: the epidermis, dermis and hypodermis^1^. The epidermis is mainly composed of keratinocytes, with melanocytes and Langerhans cells playing roles in pigmentation and immune response, respectively. The underlying dermis contains fibroblasts, blood vessels and extracellular matrix proteins that provide strength and elasticity. The hypodermis, composed of fat and connective tissue, offers insulation and energy storage.

**Fig. 1:**
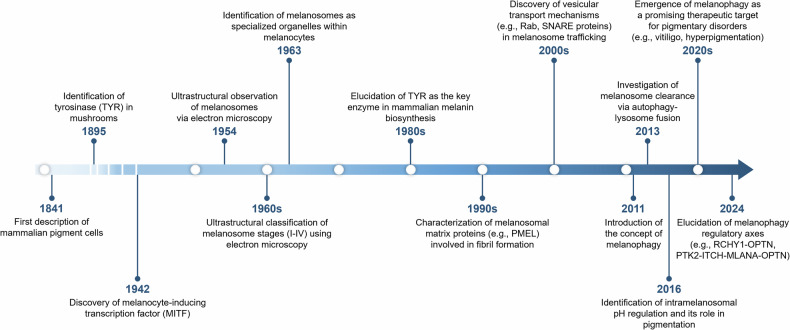
Timeline of key milestone in melanosome research. This timeline highlights pivotal advances in the study of melanosomes, from the initial morphological description of pigment cells in the 1840s to discoveries related to melanosome biogenesis, maturation and transport. Advances in the 2010s have shed light on the regulation of melanosome homeostasis. More recently, studies have begun to explore the role of melanophagy in the context of pigmentary disorders.

Melanosomes are highly specialized, lysosome-related organelles, approximately 500 nm in diameter, which reside in melanocytes^2^. These pigment granules were first noted in the 1800s but were identified as distinct organelles only in the mid-twentieth century^3,4^. In 1963, Seiji and colleagues proposed that these structures were unique to pigment-producing cells and introduced the term ‘melanosome’ to describe intracellular granules that harbor tyrosinase activity—key sites for melanin biosynthesis and storage^5–7^ (Fig. 1).

Further refined EM-based studies elaborated on the ultrastructural transitions underlying melanosome development, offering deeper insights into the molecular and cellular mechanisms governing organelle maturation and pigment deposition^6,8^. During the 1980s, tyrosinase was extensively characterized as the central melanogenic enzyme regulating melanin synthesis in mammals^9,10^, and later, studies of melanosomal matrix proteins such as premelanosome protein (PMEL/Pmel17) revealed their roles in fibril formation and melanin deposition^11^. Recent studies on Rab and SNARE proteins have revealed key vesicular pathways involved in melanosome transport and transfer to keratinocytes, with increasing evidence pointing to a role for extracellular vesicles in this process^12–14^. As the primary determinant of pigmentation in skin, hair and eyes, melanin plays a photoprotective role by shielding cells from ultraviolet (UV) irradiation and mechanical stress^15,16^. Although melanosomes share certain biogenetic and trafficking features with lysosomes, they follow a distinct, melanocyte-specific maturation process involving coordinated steps of vesicle trafficking, enzyme loading and eventual transfer to keratinocytes^2,17^. This process is tightly regulated by over a hundred molecular factors, and dysregulation in these pathways contributes to pigmentation disorders such as albinism^18^, hyperpigmentation and melanoma^16,19^.

**Fig. 2:**
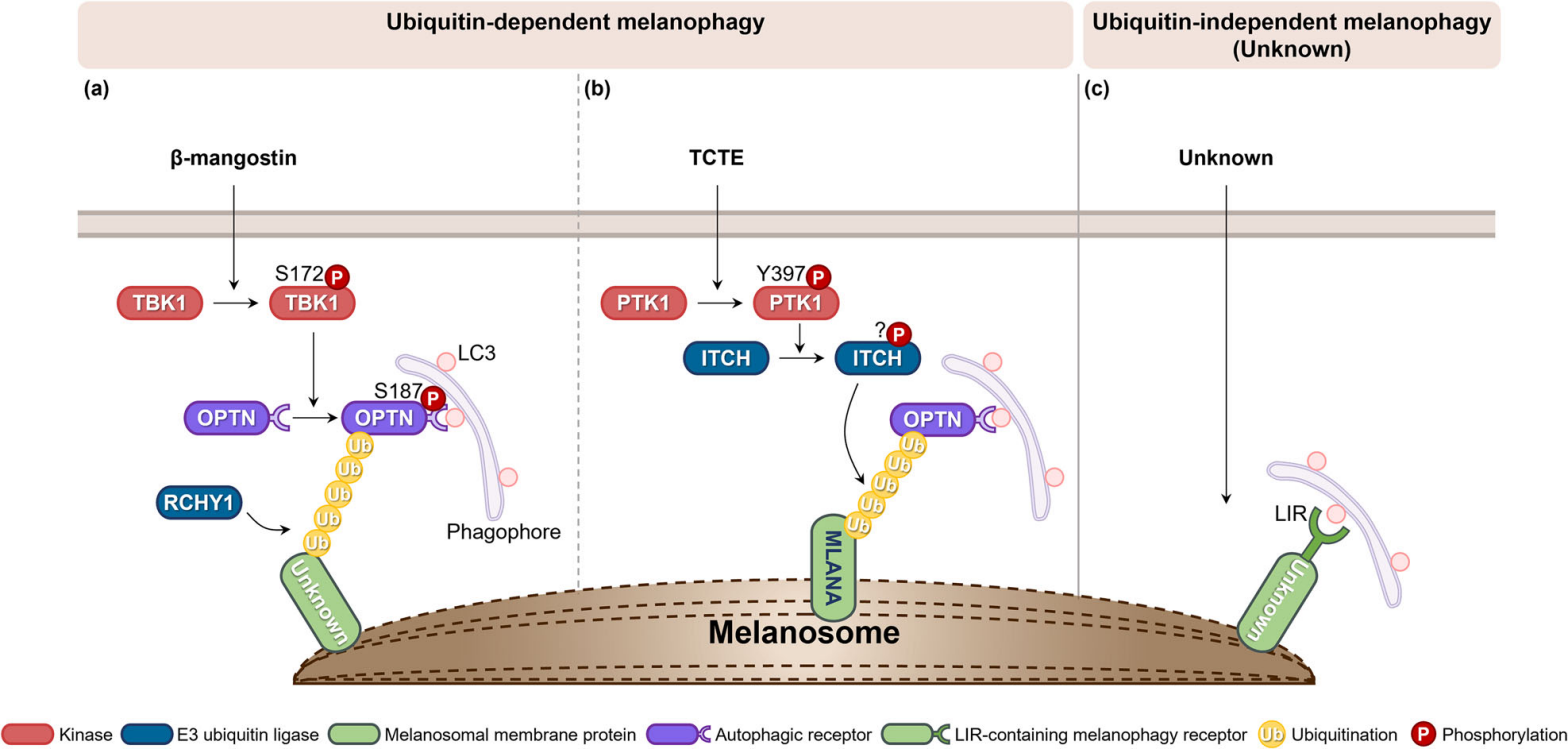
Schematic diagram of melanophagy mechanisms. This figure illustrates the current understanding and conceptual models of melanophagy regulation. Ubiquitin-dependent melanophagy involves two distinct signaling pathways. **a** In response to β-mangostin, the E3 ubiquitin ligase RCHY1 catalyzes the K63-linked polyubiquitination of melanosomal proteins, enabling recognition by the autophagic receptor OPTN. OPTN subsequently recruits TBK1, which phosphorylates OPTN at Ser187, promoting autophagosome engagement and melanophagic flux. **b** Upon TCTE treatment, PTK2 is activated via phosphorylation at Tyr397 and enhances the activity of E3 ligase ITCH. The activated ITCH ubiquitinates MLANA, which is then recognized by OPTN, facilitating lysosomal degradation of melanosomes. **c** The ubiquitin-independent melanophagy remains hypothetical. This model proposes that melanosomal membrane proteins containing LIR motifs may directly interact with autophagy machinery, bypassing ubiquitination and receptor mediation. Although such mechanisms are established in other forms of organelle-selective autophagy, they remain unconfirmed in melanophagy and warrant future investigation.

In addition to biogenesis and distribution, melanosome homeostasis relies on melanophagy, first described in detail around 2011 as a form of selective autophagy targeting melanosomes^20^. Autophagy–lysosome fusion has been shown to drive melanosome degradation and regulate pigment levels, highlighting its physiological importance^21^. In addition, receptors such as optineurin (OPTN) selectively target melanosomes, underscoring the need for precise control of melanin content^22,23^. Dysregulated melanophagy has been implicated in vitiligo, hyperpigmentation and age-related pigmentary changes and may also affect melanoma progression by helping tumor cells adapt to stress^24^. Growing evidence suggests that melanophagy is regulated by upstream factors, particularly those involving stress-responsive signaling cascades and selective autophagy receptors that mediate melanosome-specific recognition. Among these, nuclear factor erythroid-derived 2-like 2 (NRF2) has been implicated in promoting melanophagy under oxidative stress, suggesting a protective role in maintaining melanocyte homeostasis^25^. Recently, microRNAs have also been extensively studied in relation to autophagy and melanogenesis; however, their direct involvement in the selective degradation of melanosomes remains unclear and warrants further investigation^26–28^. Most recently, our group and others have reported additional melanophagy molecular regulatory pathways, including ring finger and CHY zinc finger domain-containing 1 (RCHY1)^22^ and itchy E3 ubiquitin protein ligase (ITCH)^23^ (Fig. 2).

A balanced understanding of melanosome homeostasis is important for developing better treatments for pigmentation-related conditions. Whereas traditional approaches have focused on blocking melanin production, regulating melanophagy offers a new way to manage pigment levels without interfering with other functions of melanocytes. Thus, in this Review, we highlight recent findings on how melanosomes are degraded, with a focus on melanophagy.

## Melanosome biogenesis and maturation

Melanosomes are distinct organelles involved in the production and sequestration of melanin in melanocytes. Melanosome biogenesis and maturation are fundamental to pigmentation and photoprotection. The melanosome biogenesis process is tightly regulated, integrating endosomal trafficking, protein fibrillogenesis and enzymatic melanogenesis to ensure the efficient production, storage and transfer of melanin^29^.

### Molecular mechanism for melanosome biogenesis

Melanogenesis, defined as the synthesis of melanin by melanocytes, is mainly driven by UV irradiation. This stimulation involves the generation of DNA photoproducts and the release of local signaling molecules. Among the various factors, α-melanocyte-stimulating hormone (α-MSH), secreted by keratinocytes, serves as a key inducer of melanogenesis^30^. α-MSH binds to the melanocortin 1 receptor (MC1R) on melanocytes, leading to the activation of cAMP-dependent signaling pathways. This signaling cascade ultimately enhances the expression of microphthalmia-associated transcription factor (MITF), a master regulator of melanocyte survival, proliferation and melanin production^30^. MITF transcriptionally regulates a broad spectrum of target genes essential for melanocyte function. For example, MITF drives the expression of melanosomal structural and enzymatic components, including tyrosinase, tyrosinase related protein 1 (TYRP1), TYRP2, MLANA/MART1 and PMEL^31^.

The stem cell factor (SCF)–KIT proto-oncogene, receptor tyrosine kinase (c-KIT) signaling axis is a key regulator of melanogenesis, primarily through the activation of the MAPK and PI3K pathways. The ligand-induced c-KIT autophosphorylation triggers p38 and ERK signaling, promoting CREB activation and modulating MITF levels via Ser73 phosphorylation^32,33^. Moreover, SCF–c-KIT signaling stabilizes β-catenin through PI3K-mediated GSK3β inhibition, a process further supported by Wnt signaling via Frizzled-1 and Dishevelled, enhancing MITF-driven transcription^34^.

In addition to its role in pigmentation, MITF regulates genes involved in cell cycle and survival, such as CDK2, CDKN2A and BCL2^31^. It also controls melanocyte migration via the MET proto-oncogene and supports melanosome transport by modulating RAB27A expression^35^.

### Morphological and functional maturation of melanosomes

Melanosomes originate from the endosomal system and mature through four stages, classified by ultrastructure and melanin content: unpigmented premelanosomes (stages I and II) and pigmented melanosomes (stages III and IV)^36,37^.

Stage I melanosomes arise from early endosomes and are characterized by a vacuolar appearance with a clathrin coat and limited intraluminal vesicles^38^. In stage II, melanosomes elongate and develop organized fibrillar structures primarily composed of PMEL, which provide a scaffold for melanin deposition^39,40^. Melanin synthesis begins in stage III, as enzymes such as tyrosinase, TYRP1 and TYRP2 catalyze the conversion of L-tyrosine to eumelanin or pheomelanin, depending on the context^41^. These enzymes localize to the melanosomal membrane and lumen, where additional regulators such as SLC45A2 and GRP143 modulate pH and organelle integrity^42,43^. In stage IV, dense melanin accumulation obscures internal structures, producing fully melanized, mature melanosomes^44^. Mature melanosomes are subsequently transported to the dendritic tips of melanocytes and transferred to keratinocytes, where they serve as photoprotective organelles by shielding nuclear DNA from UV damage^29^.

## Melanosome degradation

In addition to melanosome biogenesis and maturation, the regulated degradation of melanosomes is essential for maintaining melanosomal homeostasis. The accumulation of dysfunctional or surplus melanosomes can compromise cellular integrity and adversely affect melanocyte function. Melanophagy—the selective autophagic degradation of melanosomes via the autophagy–lysosome system—serves as the primary mechanism for organelle turnover^21,45^. By contrast, the ubiquitin–proteasome system primarily targets individual melanosomal proteins such as MITF or tyrosinase for degradation^46,47^. Although both systems rely on ubiquitin tagging, melanophagy uniquely facilitates the removal of entire or parts of organelles, highlighting its indispensable role in melanosomal quality control and piment regulation.

### Organelle-selective autophagy

Autophagy is a tightly controlled process that enables cells to adapt to stress, remove damaged components and maintain organelle integrity. It begins with recruitment of ATG proteins to form a phagophore, a double-membraned structure that encloses cytoplasmic material. This matures into an autophagosome, which fuses with a lysosome to form an autolysosome, where contents are degraded and recycled^48^.

Autophagosome formation is initiated by the ULK complex (ULK1/2, ATG13, FIP200 and ATG101), which is suppressed by the mechanistic target of rapamycin kinase complex 1 (mTORC1) in nutrient-rich conditions and activated during starvation^49^. Downstream, the class III PI3K complex promotes phagophore nucleation^50^. Elongation involves two conjugation systems: the ATG12–ATG5–ATG16L1 complex and ATG8 lipidation (for example, LC3 and GABARAP), catalyzed by ATG4, ATG7 and ATG3^51^. ATG9 supplies membranes for expansion^52^.

Unlike nonselective autophagy, which randomly engulfs cytoplasmic material, selective autophagy is mediated by cargo recognition mechanisms involving autophagic receptors that bridge specific substrates to the core autophagic machinery^53^. Key autophagic receptors, including sequestosome 1 (SQSTM1) and OPTN, recognize ubiquitinated cargo and facilitate its recruitment to autophagosomes through direct interactions with lipidated LC3 proteins on autophagic membrane^54^.

Selective autophagy encompasses a range of pathways targeting specific cellular components, such as mitophagy, lysophagy and pexophagy^53^. Among various forms of organelle-selective autophagy, mitophagy has been the most extensively characterized. Therefore, elucidating the molecular mechanisms underlying mitophagy provides valuable insights into the regulation of other types of organelle-selective autophagy. Selective autophagy can be categorized into ubiquitin-dependent and ubiquitin-independent pathways, based on the mechanism of cargo recognition. In the ubiquitin-dependent pathways, substrates are tagged with ubiquitin and subsequently recognized by autophagic receptors such as SQSTM1, which bind both ubiquitin and LC3, thus tethering the cargo to autophagosomes^53,55^. In particular, ubiquitin-dependent mitophagy is predominantly regulated by the PTEN-induced kinase 1 (PINK1)–parkin RBR E3 ubiquitin protein ligase (PRKN) signaling axis, which orchestrates the selective recognition and clearance of dysfunctional mitochondria^56^. Upon mitochondrial depolarization, PINK1 accumulates on the outer membrane of impaired mitochondria, where it recruits and directly phosphorylates PRKN at Ser65, leading to its activation. The activated PRKN subsequently ubiquitinates outer mitochondrial membrane proteins such as MFN2, VDAC and TOMM20^57^. These ubiquitin signals are then recognized by LC3-interacting region (LIR)-containing autophagic receptors that facilitate mitochondrial sequestration into autophagosomes^58^. However, ubiquitin-independent mitophagy relied on receptors such as BNIP3, NIX and FKBP8, which directly bind to LC3 without ubiquitin tagging^59^. Together, these parallel mechanisms ensure specificity and adaptability in targeting diverse organelles for autophagic degradation.

### Melanophagy

Melanophagy removes dysfunctional melanosomes, including immature, excess or damaged ones, in both melanocytes and keratinocytes, thereby maintaining organelle quality and pigmentation balance. When melanophagy is impaired, melanosomes accumulate abnormally, leading to pigmentation disorders such as hyperpigmentation^21,45^.

#### Melanophagy in keratinocytes

Mature melanosomes are transferred from melanocytes to surrounding keratinocytes through dendritic processes. Within keratinocytes, melanophagy plays a role in regulating epidermal pigmentation. Consistently, recent studies have revealed that the autophagic capacity of keratinocytes notably contributes to skin color variation among different ethnicities^21,60^. For instance, keratinocytes derived from lighter skin types display greater autophagic activity and show enhanced responsiveness to melanosome-induced autophagy compared with those from darker skin types^21^. This difference in autophagic potential affects the rate of melanosome degradation, ultimately determining the amount of melanin retained in the epidermis. Although these findings provide intriguing insights, they are based primarily on in vitro studies with limited donor samples. Further research involving broader populations and in vivo validation is needed to validate and expand these observations.

Further supporting evidence links autophagy to pigment regulation in keratinocytes. Functional inhibition of autophagy—either pharmacologically or via the silencing of ATGs—leads to the accumulation of melanosomes in keratinocytes, whereas the activation of autophagy reduces melanin content, as shown in both ex vivo and in vitro human skin models^29^. Various agents such as 5-methyl-3-tetradecylidene-dihydro-furan-2-one (DMF02)^25^, liensinine, neferine^61^, pentasodium tetracarboxymethyl palmitoyl 21 dipeptide 12 (PTPD-12)^60^ and even radiofrequency stimulation^62^ have been identified to enhance melanophagy and thereby exert antipigmenting effects in keratinocytes (Table 1). Moreover, epidermal autophagic activity has been shown to decline with aging, which impairs melanosome clearance and results in melanin accumulation—contributing to the development of hyperpigmented lesions such as senile lentigo^63^.

**Table 1:** List of melanophagy inducers and candidates.

	Chemicals	MoA	Ref.
**Melanophagy inducers**	2′-Fucosyllactose (2′-FL)	AMPK–ULK1 axis	^81^
	3,4,5-Trimethoxy cinnamate thymol ester (TCTE)	PTK2–ITCH–MLANA–OPTN axis	^23,45^
	3′-hydroxydaidzein (3′-ODI)	–	^72^
	3-O-Glyceryl-2-O-hexyl ascorbate (VC-HG)	Melanosome transport inhibition	^67^
	5-Methyl-3-tetradecylidene-dihydro-furan-2-one (DMF02)	NRF2–SQSTM1	^25^
	ARP101	–	^73^
	Liensinine	–	^61^
	β-mangostin	RCHY1–OPTN axis	^22,71^
	Nalfurafine hydrochloride	–	^75^
	Neferine	–	^61^
	Pentasodium tetracarboxymethyl palmitoyl 21 dipeptide-12 (PTPD-12)	–	^60^
	Picosecond laser	PI3K–AKT–mTOR	^89^
	Radiofrequency irradiation	–	^62^
	Retagliptin phosphate	–	^76^
	Resveratrol	Tyrosinase inhibitor	^64,65^
	Teneligliptin hydrobromide	–	^76^
	Ursolic acid	–	^74^
**Melanophagy-inducing candidates**	3-*O*-ethyl ascorbic acid (EAA)	NRF2–KEAP1	^90^
	7-methylsulfinylheptyl isothiocyanate (7-MSI)	ERK	^91^
	Betel leaves (*Piper betle* L.) ethanol extract	–	^92^
	Coenzyme Q0	NRF2–KEAP1–SQSTM1	^83,93^
	Ectoine	NRF2	^84^
	Ellagic acid (EA)	NRF2–KEAP1/tyrosinase inhibitor	^85^
	Hinokitiol	AKT–mTOR	^80^
	Imperatorin	PI3K–AKT	^79^
	Isoliquiritigenin	PI3K–AKT–mTOR	^77^
	Lipopolysaccharide	–	^94^
	*Patrinia villosa* (Thunb.) Juss ethanol extract (Pv-EE)	ERK	^95^
	*Panax ginseng* berry rare saponin	–	^96^
	Pterostilbene	PI3K–AKT–mTOR	^78^
	Rottlerin	–	^97^
	Schaftoside	–	^98^
	Tranexamic acid (TXA)	ERK1/2	^99^

#### Melanophagy in melanocytes

Melanophagy also occurs within melanocytes and serves as a critical mechanism for maintaining intracellular melanin homeostasis. Several keratinocyte-derived factors, such as α-MSH, are secreted in response to UV irradiation or environmental stress and stimulate melanocyte proliferation and melanogenic enzyme expression, including tyrosinase^30^. Notably, recent studies have elucidated a link between tyrosinase inhibition and the activation of melanophagy. Resveratrol exhibits both the direct enzymatic inhibition of tyrosinase and the indirect transcriptional repression of melanogenic regulators including tyrosinase, TYRP1, TYRP2 and MITF in α-MSH-stimulated melanocytes^64–66^.

Furthermore, disruption of melanosome transport can lead to their accumulation in melanocytes, triggering autophagic degradation. For example, 3-*O*-glyceryl-2-*O*-hexyl ascorbate (VC-HG) has been shown to suppress melanogenesis by activating autophagy (Table 1). It disrupts melanosome transport through the downregulation of motor proteins, such as myosin Va and kinesin, leading to melanosome accumulation and subsequent degradation via autophagy^67,68^. These findings collectively suggest that the attenuation of melanogenesis may act as a trigger for melanophagy.

Taken together, these observations underscore distinct cell type-specific regulatory mechanisms of melanophagic flux. Keratinocytes appear inherently more active in melanosome clearance—modulated by skin phototype and aging—whereas melanocytes regulate melanophagy in response to melanin synthesis status and organelle dynamics. These differences reflect the complex interplay between pigment production and degradation pathways, suggesting that interventions targeting melanophagy should consider the unique regulatory context and clearance capacities of each cell type.

Although melanophagy is essential for melanosome homeostasis, its dysregulation has been also implicated in hypopigmentary disorders^24^. In vitiligo, impaired autophagy—linked to defective NRF2–SQSTM1 signaling and reduced ATG expression—renders melanocytes more susceptible to oxidative stress and apoptosis^69^. Conversely, excessive autophagy may also contribute to melanocyte loss in stable lesions. In tuberous sclerosis complex, mTOR hyperactivation disrupts autophagic balance and reduces melanogenesis^70^. These findings suggest that both insufficient and excessive melanophagy, depending on disease context, may contribute to melanocyte dysfunction and pigmentary loss.

### Molecular mechanisms of melanophagy

Melanophagy is primarily regulated through a ubiquitin-dependent mechanism, in which melanosomal proteins are tagged with ubiquitin and recognized by selective autophagic receptors. By contrast, a potential ubiquitin-independent pathway—such as the direct interaction between LC3 and melanosomal membrane proteins—has not yet been investigated. Accordingly, this Review focuses on the current understanding of ubiquitin-mediated regulation of melanophagy, with particular emphasis on the roles of E3 ubiquitin ligases and autophagic receptors that coordinate melanosome degradation.

#### RCHY1–OPTN signaling in β-mangostin-induced melanophagy

Recent insights have revealed a ubiquitin-dependent melanophagy pathway mediated by the E3 ligase RCHY1, selective autophagic receptor OPTN and TANK binding kinase 1 (TBK1), which collectively regulate β-mangostin-induced melanosome degradation^22,71^ (Fig. 2a and Table 1). β-mangostin promotes K63-linked polyubiquitination of melanosomal proteins via RCHY1, enabling their recognition by OPTN. OPTN subsequently recruits TBK1 to melanosomes, leading to TBK1-mediated phosphorylation of OPTN at Ser187, a step for autophagosome engagement and melanophagic flux. Loss-of-function of RCHY1, OPTN or TBK1 disrupts β-mangostin-induced melanosome degradation, implicating the central role of this signaling axis in melanosomal homeostasis. Conversely, the activation of this pathway significantly downregulates melanogenic proteins such as tyrosinase and PMEL17, effects reversible by autophagy inhibitors or ATG5 depletion. Although this pathway highlights the importance of ubiquitin-mediated signaling in melanophagy, the specific melanosomal membrane substrates targeted for RCHY1-mediated ubiquitination remain to be elucidated. Together, these findings position the RCHY1–OPTN axis as a key regulator of ubiquitin-tagged melanosome clearance^22^.

#### PTK2–ITCH–MLANA–OPTN axis in TCTE-induced melanophagy

In a recent study, a ubiquitin-dependent pathway, orchestrated by the PTK2–ITCH–MLANA–OPTN regulatory axis, has been suggested as a pivotal regulator of melanophagy^23^ (Fig. 2b and Table 1). Treatment with an antimelanogenic agent 3,4,5-trimethoxycinnamate thymol ester (TCTE) primes protein tyrosine kinase 2 (PTK2) to phosphorylate the E3 ubiquitin ligase ITCH, thereby enhancing its ubiquitin-conjugating activity. The phosphorylated ITCH then ubiquitinates MLANA, a melanosomal membrane protein, tagging it for recognition by the autophagic receptor OPTN. OPTN directs the tagged melanosomes into autophagosomes, which fuse with lysosomes for the degradation of melanosomes. The inhibition or genetic ablation of PTK2 or ITCH impairs the efficiency of MLANA ubiquitination, disrupts OPTN-mediated cargo recognition and consequently reduces the overall melanophagic flux, resulting in melanosome accumulation and potential aberrant pigmentation. These findings reinforce the essential role of the PTK2–ITCH–MLANA–OPTN pathway in regulating melanophagy and maintaining pigment homeostasis.

### Melanophagy-inducing agents

A growing body of evidence suggests both established and putative small molecules can induce melanophagy, contributing to pigmentation regulation. These compounds act through a variety of mechanisms, including the direct modulation of melanophagy-specific signaling or general enhancement of autophagic activity. For clarity, we divide these agents into two categories based on the strength of mechanistic evidence supporting their role in melanophagy (Table 1).

#### Melanophagy inducers

Several natural products and small molecules have been identified as direct inducers of melanophagy with experimental evidence supporting their role in melanosome degradation via the autophagic pathways (Table 1).

Our group previously reported that compounds such as resveratrol^65^, 3′-hydroxydaidzein (3′-ODI)^72^ and ARP101^73^ inhibit α-MSH-induced melanin synthesis and promote the autophagic clearance of melanosomes by downregulating key melanogenic regulators. Ultrastructural studies with electron microscopy have corroborated these effects by demonstrating the sequestration of melanosomes within autophagosomes, indicating the role of lysosomal degradation in their depigmenting effects. Recent advances have identified melanophagy-inducing agents through functional screening approaches using fluorescent reporters of autophagic flux. For instance, TCTE (also known as Melasolv)^45^, ursolic acid^74^, nalfurafine hydrochloride^75^, teneligliptin hydrobromide and retagliptin phosphate^76^ significantly reduce melanin content by enhancing melanophagic flux (Table 1). Their effects are abolished upon autophagy inhibition or *ATG5* depletion. In addition, β-mangostin, a natural xanthone chemical, also suppresses melanogenesis by reducing the protein levels of tyrosinase and TYRP1 without affecting their transcription, probably through posttranslational regulation^71^ (Table 1). These findings underscore the biological importance of melanophagy in pigmentation regulation.

#### Melanophagy-inducing putative candidates

A second group of compounds has been reported to enhance autophagy and exert depigmenting effects, although direct evidence for melanosome selective degradation via melanophagy remains limited. These agents primarily target upstream regulators of autophagic flux and pigmentation rather than melanophagy-specific pathways (Table 1).

According to this notion, compounds such as isoliquiritigenin^77^, pterostilbene^78^, imperatorin^79^ and hinokitiol^80^ stimulate autophagic activity and exhibit notable skin-lightening effects by inhibiting the PI3K–AKT–mTOR signaling cascade, a key negative regulator of autophagy. Similarly, 2′-fucosyllactose (2′-FL) enhances autophagic flux through the activation of the AMPK–ULK1 pathway, resulting in decreased melanin accumulation in melanocytes^81^. These findings suggest that energy-sensing and nutrient-related pathways may play a role in autophagy-mediated pigment turnover (Table 1).

In addition, the SQSTM1–KEAP1–NRF2 axis has been introduced as a key regulatory hub linking autophagy and oxidative stress responses in melanosome degradation. SQSTM1 serves as a dual-function receptor, directing polyubiquitinated KEAP1 toward autophagosomes via LC3 binding, thus relieving KEAP1-mediated repression of NRF2. Activated NRF2 subsequently translocates to the nucleus and upregulates antioxidant genes, including HO-1^82^. Agents such as VC-HG^67^, coenzyme Q0^83^, ectoine^84^ and ellagic acid^85^ have been shown to activate this pathway, contributing to autophagic activation and depigmentation (Table 1).

Although these agents engage signaling pathways that are mechanistically linked to autophagy and melanogenesis regulation, whether their effects are mediated specifically through melanophagy remains to be fully established. Further studies are warranted to determine whether these compounds promote the selective degradation of melanosomes or exert broader effects on other organelles and cellular processes.

## Potential applications of melanophagy

Pigmentation homeostasis relies on a precise balance between melanosome biogenesis, maturation, transport and degradation. Notably, conventional cosmetic strategies primarily target melanin production by inhibiting enzymes such as tyrosinase and MITF. Although they are effective in suppressing melanin synthesis, these approaches frequently cause the accumulation of dysfunctional, immature stage III melanosomes, characterized by incomplete melanization and compromised cellular functions^86,87^. These impaired and dysfunctional melanosomes cannot properly mature or transfer to keratinocytes, necessitating their removal to avoid cellular stress and persistent pigmentation. As we discussed, melanophagy has emerged as a crucial mechanism addressing this challenge. Specifically, melanophagy degrades immature, dysfunctional or excessively produced melanosomes in melanocytes and keratinocytes. Failure to effectively clear these melanosomes results in intracellular accumulation, disturbing pigment homeostasis and potentially exacerbating hyperpigmentation. Thus, the activation of melanophagy offers a promising approach to promote natural pigment turnover for both medical and cosmetic applications. According to this notion, recent studies highlight promising cosmetic compounds such as TCTE^45^ and resveratrol^65^, which stimulate melanophagy, reducing intracellular melanin effectively. Although these agents highlight the potential of targeting melanophagy, current evidence is largely limited to in vitro and preclinical models, and clinical validation remains lacking. Whereas melanophagy provides substrates specificity, the nonspecific activation of autophagy pathways may carry the risks, including the unintended degradation of essential organelles and disruption of cellular homeostasis. These concerns underscore the need for a deeper mechanistic understanding of melanophagy-specific regulation to enable safe and effective therapeutic strategies.

Integrating melanophagy activators with conventional melanin synthesis inhibitors may offer a dual-action strategy, simultaneously blocking pigment formation and accelerating the clearance of existing dysfunctional melanosomes. Such combined formulations could provide superior and sustained skin-brightening outcomes, addressing limitations of traditional agents that solely inhibit pigment production. Moreover, melanophagy-targeted products align with current customer trends favoring balanced, holistic skin health rather than aggressive skin-whitening strategies. To maximize both safety and efficacy, future development should prioritize delivery systems that enable skin-specific or cell type-specific activation of melanophagy pathways. Topical formulations utilizing nanocarriers, liposomes, or prodrug-based strategies may help localize activity to melanocytes or keratinocytes, thereby minimizing systemic exposure and reducing the risk of off-target effects. Advances in such precision delivery technologies will be essential for translating melanophagy from a mechanistic insight into a viable therapeutic or cosmetic modality.

## Perspectives on melanophagy

Melanophagy is emerging as an essential mechanism in regulating skin pigmentation, with promising implications beyond cosmetic applications. However, several critical research questions remain. Mechanistically, current studies have focused exclusively on ubiquitin-dependent melanophagy, which involves ITCH or RCHY1. By contrast, ubiquitin-independent pathways, which are well-documented in other forms of selective autophagy such as mitophagy and ER-phagy (selective autophagy of the ER), remain uncharacterized in the context of melanosome degradation. Thus, additional studies are needed to elucidate these alternative mechanisms.

Future studies should investigate the regulation of melanophagy across different skin types and its crosstalk with other cellular degradation pathways. Understanding how stressors such as aging, inflammation and metabolic imbalance influence melanophagy may further uncover its therapeutic relevance. Clinically, melanophagy is considered as a promising target for both cosmetic enhancement and the treatment of pigmentation disorders, including vitiligo, melasma, postinflammatory hyperpigmentation and age-related pigmentary changes. To translate these insights into practice, the further elucidation of its molecular mechanisms, the identification of specific modulators and the development of reliable in vivo models and noninvasive monitoring tools will be essential.

Beyond pigmentation, the role of melanophagy in other pathological conditions, such as melanoma, is emerging but remains poorly understood. Whereas autophagy can both suppress tumor initiation and support tumor progression^88^, it is unclear whether melanophagy follows a similar context-dependent pattern. Melanosomes themselves may also exert pro- or antitumorigenic effects through their roles in redox balance and pigment metabolism. Thus, melanophagy could either enhance tumor resilience or increase the susceptibility to stress. Further investigation may uncover new therapeutic targets for tumor adaptation and resistance.

In conclusion, advancing melanophagy research may enable the development of next-generation cosmetic solutions for pigmentation control, contributing to healthier and more balanced skin.
